# Physical Mechanism of Photoinduced Charge Transfer in One- and Two-Photon Absorption in D-D-π-A Systems

**DOI:** 10.3390/ma14143925

**Published:** 2021-07-14

**Authors:** Xinyue Wang, Di Wang, Jia Li, Meixia Zhang, Peng Song

**Affiliations:** College of Physics, Liaoning University, Shenyang 110036, China; wxy241227@163.com (X.W.); wd982846113@163.com (D.W.); lijia@lnu.edu.cn (J.L.)

**Keywords:** one-photon absorption, two-photon absorption, absorption cross-section, charge transfer

## Abstract

The photoinduced charge transfer process of a D-π-A molecule (W1) and three D-D-π-A molecules (WS5–WS7) with triphenylamine as a donor was studied theoretically. D-D-π-A molecules are formed by inserting donors between the triphenylamine and π-linker (π-bridge) on the base of the W1 molecule. The results showed that donor insertion resulted in a red shift in the absorption spectrum, and the absorption intensity increased to a certain extent. A visualization method was used to observe the charge transfer of the four molecules in the process of one- and two-photon absorption (TPA). The local excitation enhanced charge transfer excitation in the TPA process was analyzed and discussed, and the insertion of the thiazolo[5,4-d]thiazole donor showed the largest TPA cross-section. This work contributed to the profound understanding of D-D-π-A molecules and the design of large cross-section TPA molecules.

## 1. Introduction

Photoinduced charge transfer not only occurs in one-photon absorption (OPA) but also occurs in two-photon absorption (TPA), which is an electronic excitation phenomenon. TPA refers to the transition of an atom or molecule to a higher energy level by simultaneously absorbing and combining two low-energy photons, i.e., the total energy of the two photons is close to that of a stable excited molecule state, which is a third-order nonlinear process [1]. As early as 1931, Göppert-Mayer first applied the second-order perturbation theory to carry out theoretical research on TPA [2]. In 1961, Kaiser and Garret first proved the existence of this nonlinear optical phenomenon experimentally [3]. Compared to OPA, TPA has a longer excitation light wavelength and a stronger light wave penetration ability. The probability of TPA is positively correlated with the square of the emitted light intensity [1], so it has strong spatial selectivity and high information storage density. Therefore, it has a wide range of applications in two-photon fluorescence microscopy [4,5,6,7], three-dimensional (3D) optical data storage [8,9,10], photodynamic therapy [11,12,13,14,15], and dye-sensitized solar cells [16,17,18,19].

The size of TPA is determined by its cross-section. It has been shown that linking the electron donor (D) to the electron acceptor (A) through a π-conjugated bridge and increasing the intensity and effective length of the π-conjugated bridge [1,20,21] result in a larger TPA cross-section. Simultaneously, when the intensities of the D and A are properly adjusted, the TPA is also strengthened. At present, there are A-π-D-π-A, D-π-A-π-D, and D-D-π-A [22,23,24,25] strategies for increasing TPA.

It is well-known that charge transfer is closely related to the optical properties of molecular systems during the dynamic process of molecular excitation. The charge transfer of a TPA process may be very different from that of a OPA process, and the main factor that affects the two-photon absorption performance is the intensity of intramolecular charge transfer. Based on the sum-over-states (SOS) model, TPA includes two absorption processes, and it is necessary to study the charge transfer of both [26,27]. The two processes of TPA can be calculated using a program developed by Mu and Sun [28]. If the reciprocating charge motion occurs in the two-step transition process, the SOS method can accurately analyze the charge transfer characteristics in the two-step transition process of TPA. Their two-dimensional and 3D visualization software can be used to draw the transition density matrix (TDM) and charge density difference (CDD) of the two processes, which can directly reflect the charge transfer between each atom or fragment in the molecule [26,27,28]. This visualization of the charge transfer makes it easier to observe charge transfer patterns and analyze the properties of excited states.

In recent years, triphenylamine has been used in many applications because of its effective electron-feeding ability, hole transport performance, and the fact that the structure of its propeller reduces the probability of electron recombination by a large extent. For example, as a strong electron-donating group, triphenylamine can be connected with naphthalimide to increase its fluorescence quantum efficiency as a fluorescent probe to rapidly detect thiophene in aqueous solutions [29]. Vinyl triphenylamine compounds have a high TPA cross-section [30,31], which promotes the application of triphenylamine in two-photon applications. Recently, two compounds, TP2Py and TP2(3)Bzim, have been found to trigger cell death in response to one or two-photon excitation, which can yield promising results in two-photon photodynamic therapy [32]. Furthermore, the applications of triphenylamine in quantum dot light-emitting diodes [33] and dye-sensitized solar cells [34] should not be underestimated.

**Figure 1:**
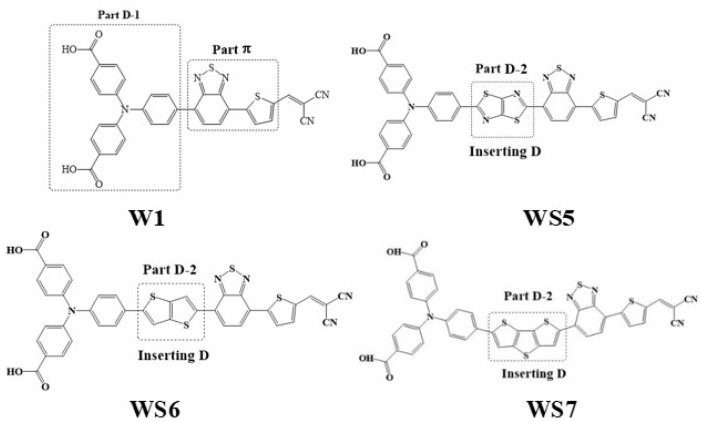
Structures of W1, WS5, WS6, and WS7 molecules.

In this study, based on the work of Wu et al. [35], the selected basic molecule was the synthesized triphenylamine-based dye W1, which is a D-π-A molecule with triphenylamine as the D, a 2,1,3-benzothiadiazole moiety as the π bridge, and dicyanovinyl as the A. The other three molecules, WS5, WS6, and WS7, are D-D-π-A molecules formed by inserting D molecules between triphenylamine and the π-linking group. The inserted D molecules included thiazolo[5,4-d]thiazole, bithiophene, and dithieno[3,2-B:2′, 3′-d]thiophene (s-DTT). The structures of W1, WS5, WS6, and WS7 are shown in Figure 1. Our calculations showed that D insertion increased the molecular absorption intensity, reduced the molecular energy gap, redshifted the absorption spectrum, and showed a relatively large TPA cross-section for all four molecules. The insertion of an additional D to form a D-D-π-A structure increases the molecular conjugate surface and electron donating ability and promotes intramolecular charge transfer, thereby obtaining a large TPA cross-section. In addition, a visual study of the charge excitation mode in the TPA process was carried out, and a local excitation enhanced transfer excitation occurred in the molecule.

## 2. Methodology

### 2.1. Calculation Details

In this work, Gaussian 09 D.01 software [36] was used to complete all of the quantum chemical calculations. The B3LYP functional, 6-31G(d) basis set, and density functional theory (DFT) were used to optimize the geometry of the four molecules, complete with the SMD solvent model in trichloromethane. The indicators can be found below the convergence limit. At this level, the optimized structure was analyzed using its vibration frequency, and its geometric structure was determined to be the minimal point structure [37,38,39,40]. The ability of B3LYP to calculate charge transfer excitation is relatively poor; therefore, in order to calculate the electronic transitions and absorption spectra of the four molecules, time-dependent DFT was used at the CAM-B3LYP level with long-range-correction functions and 6-311G (d, p) [41,42,43,44,45], which calculated 80 excited states, which provided reasonable results for the SOS method. On the basis of these results, the two-photon transition dipole moment integral was calculated, and the TPA spectrum was simulated [28]. The SOS method used in the calculation of TPA has been proven to be effective by comparing the results with those of the quadratic response method [46]. The TDM and CDD of the excited states and between the excited states were obtained using the multiwfn3.7 program [47], and the CDD was then plotted using VMD [48] to visualize the charge transfer during the transition process.

### 2.2. One-Photon Absorption

The oscillation strength can be used to measure the transition strength of OPA, which can be expressed as:

(1) $$f_{ij}=\frac{2w_{ij}}{3}{\left|<i\left|\mu \right|j>\right|}^{2}$$

where w is the transition energy between two electronic states, <i|μ|j> is the electric dipole moment between two electronic states.

### 2.3. Two-Photon Absorption

According to the SOS model, TPA involves two absorption processes. First, the ground state molecule is converted into an excited state and is then excited to a higher excited state in the second process. The absorption cross-section of the TPA process is expressed as [27,49,50]:

(2) $$\sigma_{tp}=\frac{4\pi^{2}a_{0}^{5}\alpha }{15c_{0}}\frac{w^{2}g\left(w\right)}{\Gamma_{f}}\delta_{tp}$$

where $c_{0}$ is the speed of light, $\Gamma_{f}$ is the material fine structure constant, $a_{0}$ is the Bohr radius, and g(w) is the profile of the spectral line and is a function of the transition probability, $\delta_{tp}$, which is expressed as:

(3) $$\delta_{tp}=8\underset{\begin{array}{c} j\neq g\\ j\neq f \end{array}}{\sum }\frac{{\left|<f\left|\mu \right|j>\right|}^{2}{\left|<j\left|\mu \right|g>\right|}^{2}}{{\left(w_{j}-w_{f}/2\right)}^{2}+\Gamma_{f}^{2}}\left(1+2\cos^{2}\theta_{j}\right)+8\frac{{\left|\Delta \mu_{fg}\right|}^{2}{\left|<f\left|\mu \right|g>\right|}^{2}}{{\left(w_{f}/2\right)}^{2}+\Gamma_{f}^{2}}\left(1+2\cos^{2}\phi \right)$$

where g represents the ground state, j represents the intermediate state, f represents the final state, |g⟩, |j⟩, and |f⟩ are the wavefunctions of the three states, and <f|μ|j> and <j|μ|g> are the transition dipole moments of the two processes, respectively, with an angle of $\theta_{j}$ between them. The permanent dipole moments of the g and f states are expressed as <g|μ|g> and <f|μ|f>, respectively, and $\Delta \mu_{fg}$ is the difference between the two. ϕ is the angle between $\Delta \mu_{\mathrm{fg}}$ and <f|μ|g>, and $\Gamma_{f}$ is the lifetime of the f state. The first part of the Equation (3) represents the contribution of a two-step transition to two-photon absorption probability, that is, the transition from the ground state to the intermediate state and then from the intermediate state to the final state. As such, an intermediate state is experienced. Therefore, this part is called the “three-state” term. The other part of Equation (3) is the contribution of a one-step transition to two-photon absorption probability without an intermediate state, that is, a one-step transition from the ground state to the final state; thus, this part is called the “two-state” term.

## 3. Results and Discussion

### 3.1. OPA and TPA Spectra

**Figure 2:**
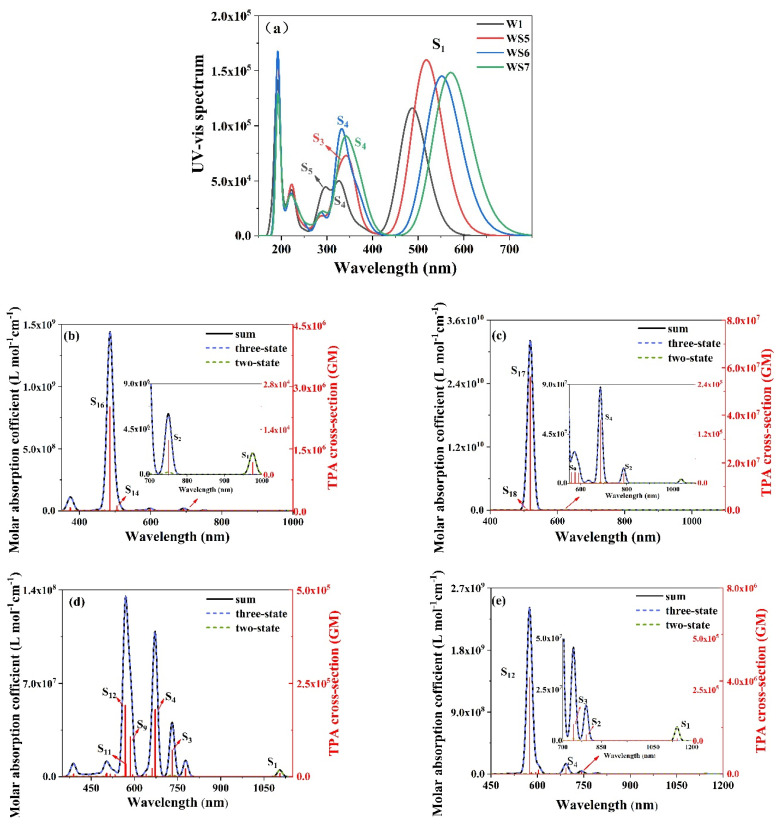
OPA UV-vis (**a**) and TPA spectra (**b**–**e**). The gray, red, blue, and green lines represent W1, WS5, WS6, and WS7, respectively. The black, blue, and green lines in (**b**–**e**) represent the sum, three-state, and two-state terms, respectively.

The OPA and TPA simulated spectra of W1, WS5, WS6, and WS7 are shown in Figure 2. Figure 2a shows the simulated UV-Vis spectrum of the four molecules using Gaussian broadening (the broadening parameter is 0.37 eV), and it can be seen that the spectral line shapes of these four molecules were very similar. The lowest energy absorption peak, namely, the strongest absorption peak, was contributed to by the excited state of S_1_, which was determined by the intensity of the oscillator. The excitation energy and oscillator intensity of the S_1_ excited state for the four molecules are shown in Table 1. From W1 to WS7, the corresponding wavelengths of the lowest energy absorption peaks contributed by the excited state of S_1_ were 486.86 nm (1—black), 517.86 nm (2—red), 551.58 nm (3—blue), and 551.58 nm (3—green), respectively, and their OPA spectra were gradually redshifted, as shown in Table 1. The absorption peak of the S_1_ excited state of WS7 produced the greatest red shift, and its absorption range was wider. The four molecules all had weak absorption peaks at 220–400 nm, which mainly arose from the excited states of S_3_ to S_6_. The molar absorption coefficients of WS5, WS6, and WS7 were all greater than those of the prototype W1, indicating that the insertion of the D increased the conjugate surface, which not only made the absorption spectrum redshift, but also increased the light absorption intensity.

Figure 2b–e show the TPA spectrum obtained by linearly broadening the TPA cross section of the four molecules (the broadening parameter is 0.617 eV). The left axis is the TPA molar absorption coefficient, and the right axis is the TPA cross-section. As it can be seen, the spectral shape of W1 was very similar to that of WS5 and WS7, which had one of the strongest absorption peaks and weak absorption peaks around it. From the TPA spectra of WS5, WS6, and WS7, it can be seen that with the insertion of different donors, the TPA peak gradually redshifted and its trend was the same as that for the OPA spectra. The TPA spectrum of WS6 was different from that of the other three molecules. The distribution of its absorption peaks was much more complex, and there were many absorption peaks, but the strongest absorption peak of WS6 was also contributed to by S_12_, as was also the case for WS7.

Compared with the prototype W1, the TPA cross-sections of WS5 and WS7 both increased to a certain extent, while the cross-section of WS6 was smaller than that of W1, however, its absorption range was the largest. According to Equation (2), the TPA cross section is the sum of the two-step transition and the one-step transition (black line). The blue line represents the contribution of the “three-state” term of the two-step transition to the two-photon absorption probability, and the green line represents the contribution of the “two-state” term of the one-step transition to the two-photon absorption probability. With the exception of the S_1_ excited state, the black and blue lines were highly fitted, indicating that the “three-state” term was dominant. For S_1_, it can be seen that the “two-state” term dominated and was contributed to by the permanent dipole moments of the initial and final states.

### 3.2. Frontier Molecular Orbitals

**Figure 3:**
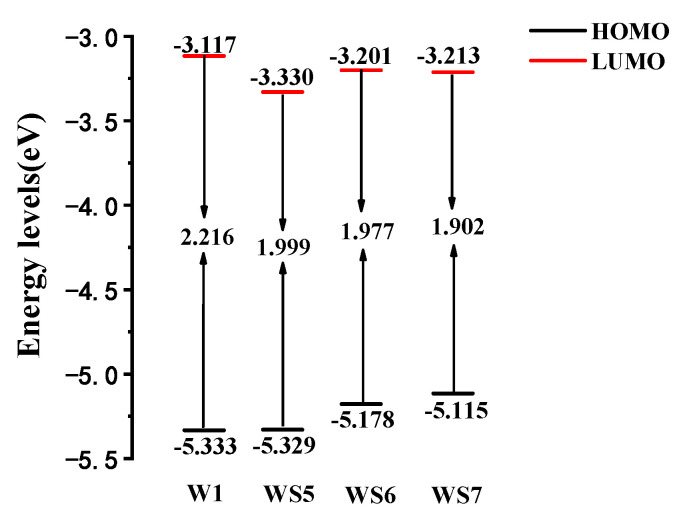
Frontier molecular orbital energy level diagrams of W1, WS5, WS6, and WS7.

Figure 3 shows the energies of the molecular frontier orbitals of the four molecules in the optimized ground state structure, including the energy of the highest occupied molecular orbital (HOMO), the lowest unoccupied molecular orbital (LUMO), and the energy gap between the two. The energy gaps of WS5, WS6, and WS7 were all smaller than those of the prototype W1, indicating that D insertion was conducive to reducing the HOMO–LUMO gap. The reduction of the energy gap helped to absorb light with longer wavelengths. The gap from WS5 to WS7 decreased gradually, with the energy gap of WS7 being the smallest. Therefore, the insertion of the 2nd donor group in WS7 had the greatest impact on the reduction of the energy gap, and the lowest energy absorption peak generated the largest red shift, which is consistent with the OPA spectrum. 

**Figure 4:**
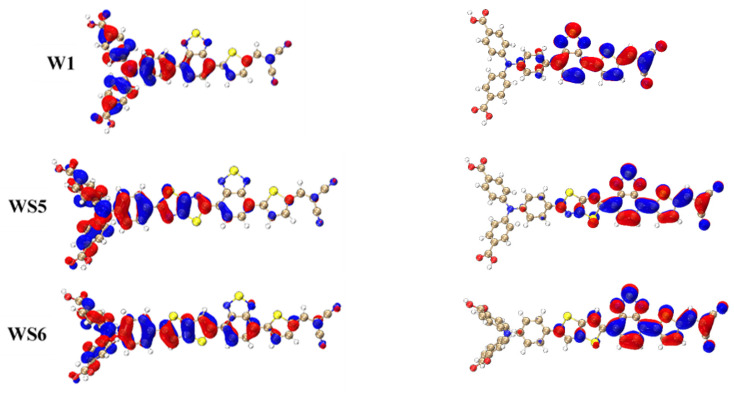

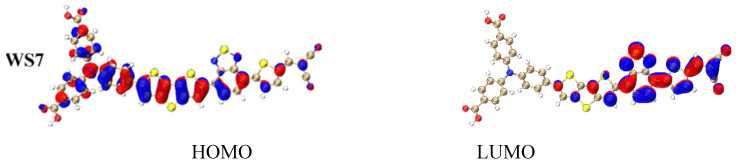
Geometrically optimized structures of W1, WS5, WS6, and WS7, and the electron density distributions of their HOMOs and LUMOs.

Figure 4 shows the frontier molecular orbitals of the four molecules. The HOMO was mainly concentrated on triphenylamine and the inserted D, with only a small amount distributed on the π-linking group. In contrast, the LUMO distribution area was the A and π-linker. For WS5, WS6, and WS7, the LUMOs were distributed on the inserted D in small amounts. WS5 was slightly different from the other three molecules in that its HOMO was less distributed on the π- linker. The small overlap between the HOMO and LUMO effectively promoted intramolecular charge transfer.

### 3.3. One-Photon Absorption

**Figure 5:**
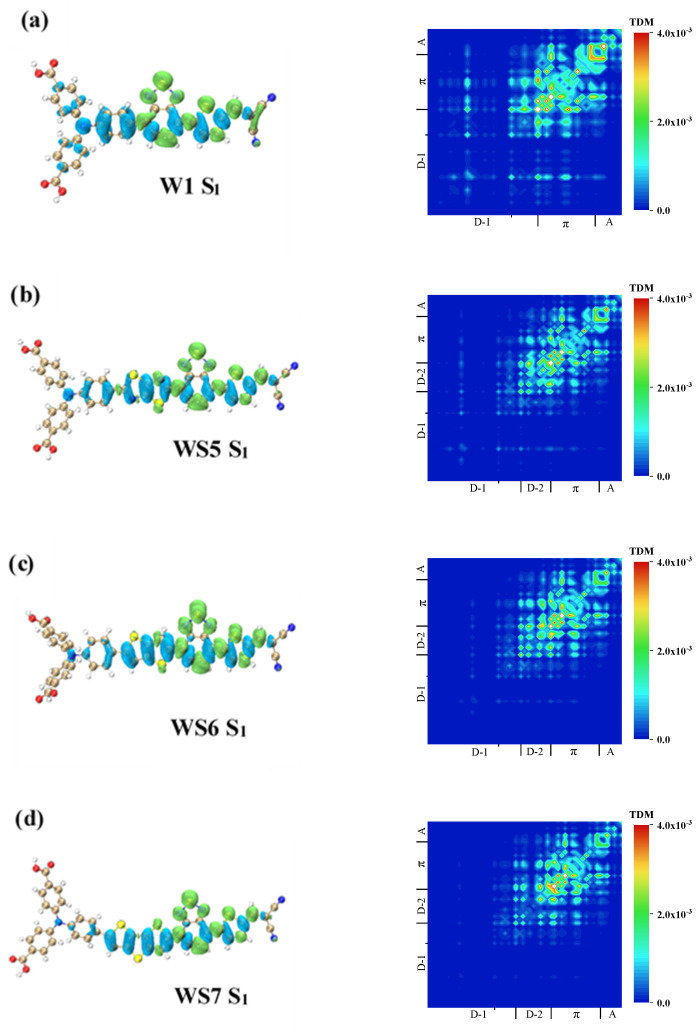
TDM and CDD of S_1_ of (**a**) W1, (**b**) WS5, (**c**) WS6, and (**d**) WS7 in OPA process. The blue isosurface represents holes, and the green isosurface represents electrons.

**Figure 6:**
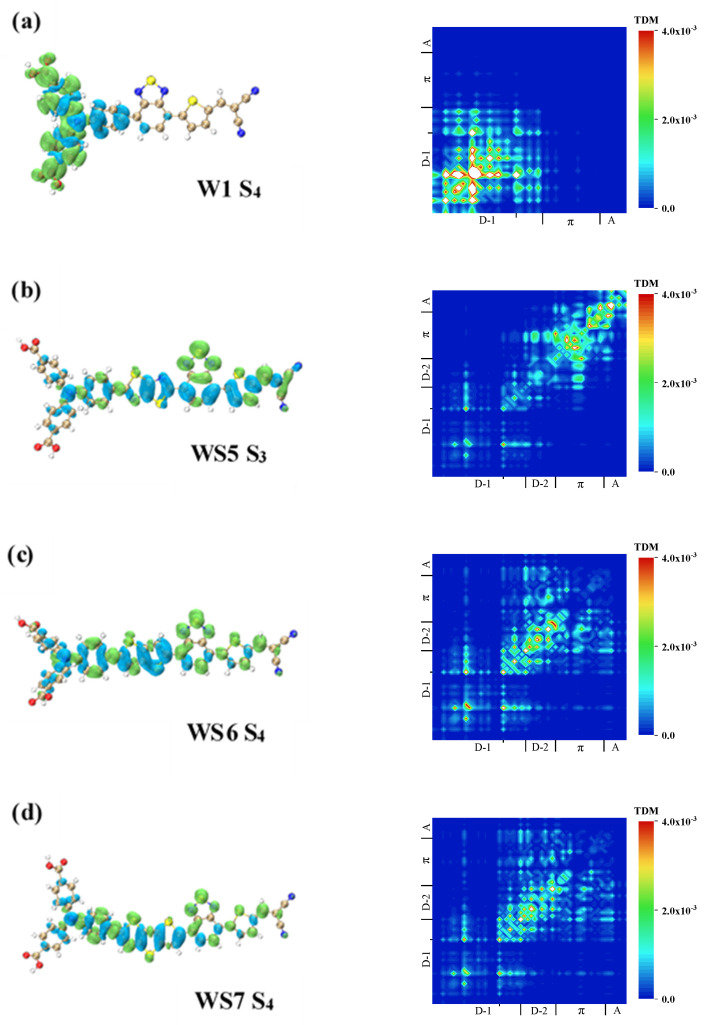
TDM and CDD of (**a**) S_4_ of W1, (**b**) S_3_ of WS5, (**c**) S_4_ of WS6, and (**d**) S_4_ of WS7 in the OPA process.

The insertion of different D molecules resulted in differences in the OPA and TPA spectra of the four molecules. Therefore, the one-photon excitation modes of these four molecules were analyzed by plotting the TDM and CDD, as shown in Figure 5 and Figure 6. The electron excitation mode of the valence layer can be divided into local excitation, which did not change the electron distribution region before and after excitation, and charge transfer excitation, which had an obvious transfer in the distribution region. Charge transfer could occur within or between molecules. The four molecules that were analyzed in this study were all molecular monomers, so they undergo intramolecular charge transfer. 

It can be seen from the OPA spectrum that S_1_ was the excited state with the highest oscillation intensity of the four molecules, so the excited state of S_1_ was first analyzed. The CDD and TDM of S_1_ of the four molecules are plotted in Figure 5. In the CDD, the blue isosurface represents the reduction of electrons (holes), and the green isosurface represents the increase of electrons (electrons). Figure 5a shows the excited S_1_ state of the W1 molecule. It can be seen from the CDD that holes were only distributed on the triphenylamine, indicating that charge transfer occurred at this part of the molecule. The distribution of electrons on the π-linker and A indicated that the charge was transferred from the triphenylamine to that part of the molecule, which also had a hole distribution, and therefore local excitation had occurred. The blue isosurface on the benzene ring attached to the π-linker was particularly large, indicating the strong charge transfer at this location. In the TDM diagram, if the absolute value of the diagonal matrix element was large, then local excitation had occurred at this part. In contrast, if the absolute value of the non-diagonal matrix element was large, charge transfer excitation had occurred at both parts. It can also be proven from the TDM that the π-linker and A showed local excitation characteristics, while the charge transfer excitation characteristics on triphenylamine were relatively weak.

The CDD and TDM of S_1_ for WS5, WS6, and WS7 are shown in Figure 5b–d, respectively. Comparison of the CDD of the three molecules showed that strong local excitation occurred on the π-linker and the A. The distribution of the holes on the inserted D indicated that charge transfer had occurred and that the direction of the charge transfer was from the D to the A. WS7 differed from the other two molecules in that hole distribution on triphenylamine was relatively small, indicating that triphenylamine had little involvement in charge transfer, see Figure 5d. It can be seen from the TDM that the charge transfer excitation characteristic of triphenylamine was indeed very weak, and the inserted D and π-linker were mainly involved in the charge transfer. The excitation modes of the S_1_ excited state of these four molecules were similar and all showed localized excitation with charge transfer.

The OPA spectra of the four molecules showed several weak absorption peaks in the wavelength range of 200–400 nm. These absorption peaks were mainly contributed to by the excited states from S_3_ to S_6_. Although the intensity of the oscillators in these excited states was relatively weak, it may be the intermediate state that realizes the two-photon transition. As for the S_4_ excited state of W1, it can be seen from the TDM in Figure 6a that local excitation occurred on triphenylamine, and the charge transfer between the two benzene rings on the outer side and the benzene ring on the inner side occurred. The CDD showed that electrons were transferred from the benzene ring on the inner side to the two benzene rings on the outer side. 

The local excitation characteristics of the S_3_ excited state of WS5 were more obvious than charge transfer excitation. It can be seen from the TDM in Figure 6b that local excitation occurred in all four parts of the molecule, and there was charge transfer between each part. The CDD showed that the π-linker electrons were partly derived from the benzene ring on the inner side of the triphenylamine and partly from the inserted D (thiazolo[5,4-d]thiazole).

The excited state of the S_3_ of WS6 was similar to the excited mode of the S_4_ of WS7. First, it can be seen in Figure 6c,d that charge transfer occurred between the benzene ring on the inner side and the outer side of triphenylamine. Second, there was strong local excitation on the inserted D, and there was charge transfer to the A. For the π-linker, 2,1,3-benzothiadiazole, the holes were mainly distributed on the benzene ring, and the electrons were mainly distributed on the 1,3,4-thiadiazole, so in the case of the group itself, the electrons were transferred from the benzene ring to the 1,3,4-thiadiazole.

### 3.4. Transition Dipole Moments

The absorption peak corresponding to electron excitation was determined by the vibrator intensity, which was positively correlated with the square of the transition electric dipole moment and, of course, to the transition energy between the two states, as shown in Equation (1). Therefore, the transition dipole moment of the whole molecule was first analyzed, as shown in Table 2. The value of WS7 was the largest but was not significantly different from that of WS5. Moreover, the transition energy of WS5 was much larger than that of WS7, so the oscillator intensity of WS5 was the largest, and the absorption peak of its excited state S_1_ in the OPA process was the strongest.

**Figure 7:**
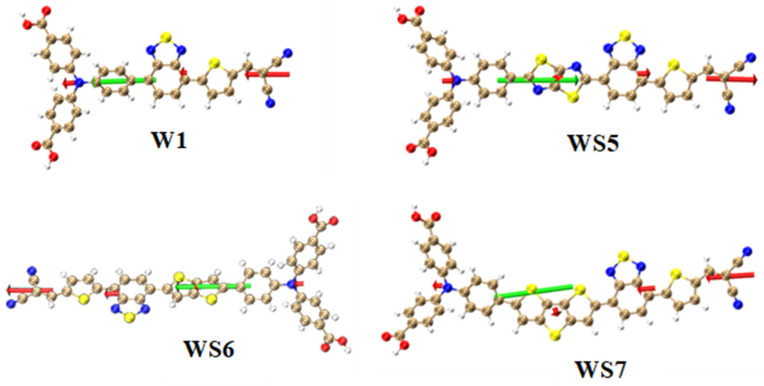
Transitional dipole moments of W1, WS5, WS6, and WS7. The red arrows represent the contribution of the fragments to the transition dipole moment, and the green arrows represent the total transition dipole moment of the molecule.

In order to analyze the contribution of different molecular fragments to the transition dipole moment of the excited state S_1_, W1 was divided into three parts, while WS5, WS6, and WS7 were divided into four parts. Their fragments are the same as those of the TDM and CDD, as shown in Table 3 and Figure 7. In Figure 7, the green arrow indicates the total transition dipole moment of the system, and the red arrow indicates the dipole moment of the corresponding part. The length and direction of the arrow correspond to the magnitude and direction of the transition dipole moment. The center of the arrow is the geometric center of the segment.

As it can be seen from Table 3 and Figure 7, first, among the four molecules, the value of the receptor transition dipole moment was the largest. The value of the receptor part of WS5 was the largest one, but the value of this part was not significantly greater than that of WS7. Although the value of the triphenylamine part of WS5 was greater than that of WS7, the inserted thiazolo[5,4-d]thiazole D, and π-linking group values were smaller than that of WS7, and the value of thiazolo[5,4-d]thiazole was the least compared to those of the inserted three donors, so the transition dipole moment of WS5 was slightly smaller than that of WS7.

### 3.5. Two-Photon Absorption

**Figure 8:**
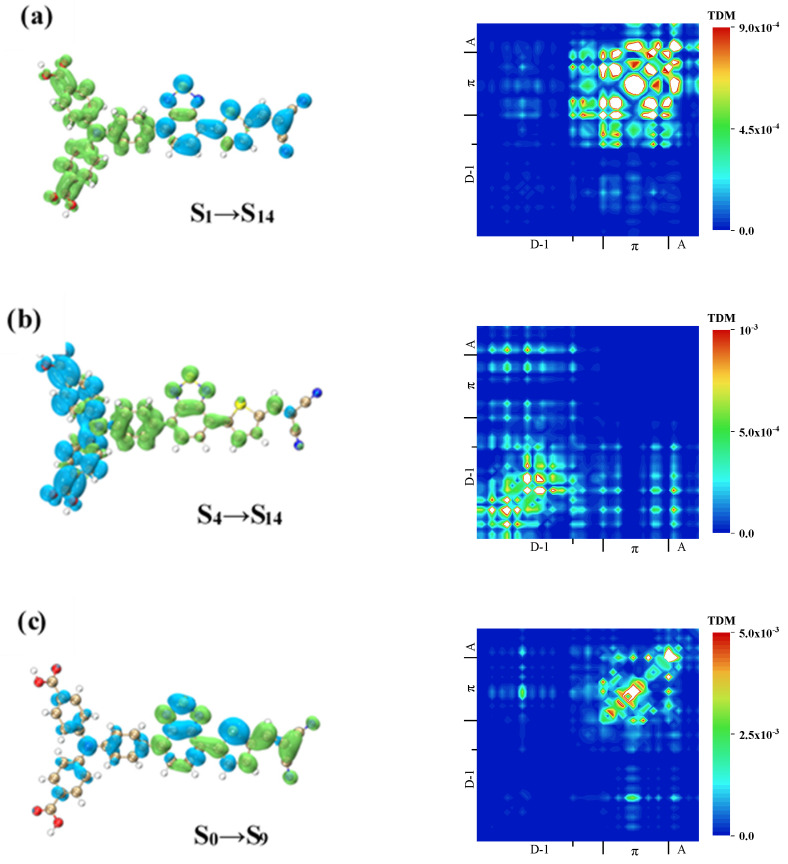

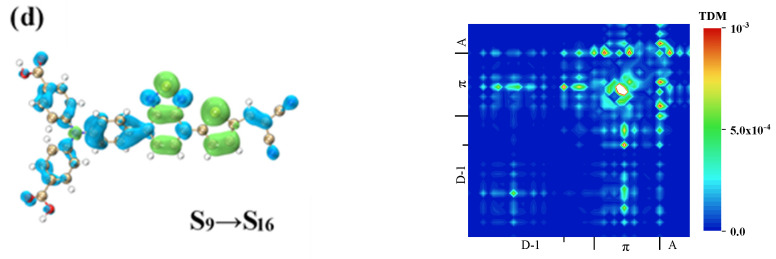
TDM and CDD of W1 in TPA. TDM and CDD of S_1_→S_14_ (**a**) of W1, TDM and CDD of S_4_→S_14_ (**b**) of W1, TDM and CDD of S_0_→S_9_ (**c**) of W1, TDM and CDD of S_9_→S_16_ (**d**) of W1.

The TPA spectra of W1, WS5, WS6, and WS7 were calculated, and the results showed that the four molecules were mainly in “three states”, i.e., the TPA process went through an intermediate process. This intermediate process was not unique, and the TPA probability that we obtained was the sum of all of the possibilities. The emergence of a TPA peak required a high probability of both processes (transition dipole moment), as shown in Table 4. We analyzed the charge transfer mode of the TPA process by drawing the TDM and CDD of the two processes. For W1, the two-photon excited states with large absorption cross-sections were S_16_ and S_14_, as shown in Figure 8, and the intermediate states with a large transition probability of S_14_ were S_1_ and S_4_. Table 4 shows the elements of the transition dipole moment matrix in TPA.

The first channel of S_14_ was analyzed, and the first step was S_0_→S_1_. According to the previous analysis, this was a local excitation with weak charge transfer. In the second step, S_1_→S_14_, as shown in the CDD in Figure 8a, the electrons were transferred from triphenylamine to the π-linker and A. TDM showed that a relatively strong local excitation occurred on the π-linker, and this resulted in a charge transfer with both the A and triphenylamine, with electronics only existing on the A, indicating that the charge transfer went from the A to the π-linker and then to the triphenylamine. Generally speaking, the charge transfer directions of these two processes were opposite, indicating that the excited state of S_14_ with S_1_ as the intermediate state was a local excited state.

The first difference in the second path was that S_0_→S_4_ was a local excitation on triphenylamine. From S_4_ to S_14_, it can be seen from the CDD in Figure 8b that electrons transferred from the two benzene rings outside the triphenylamine in the direction of the A, which can be proven by TDM. This excitation mode was the result of the local excitation of triphenylamine providing the conditional basis for the charge transfer to be carried out in the next step, so it was a local excitation enhanced charge transfer excitation.

The excited state that contributed the most to the two-photon main absorption peak of W1 was S_16,_ and the intermediate state with the highest transition probability was S_9_. From the TDM in Figure 8c, it can be seen that in the first step, S_0_→S_9_, local excitation of the π-linker occurred, and charge transfer also existed between the π-linker and A, and between triphenylamine and both the π-linker and A. The CDD demonstrated that the charge transfer direction was from triphenylamine to the A. The TDM and CDD of the second step are shown in Figure 8d. By observing the CDD, the difference from the first step was the distribution of the holes on the A, indicating the transfer of electrons from the triphenylamine and the A to the π-linker.

**Figure 9:**
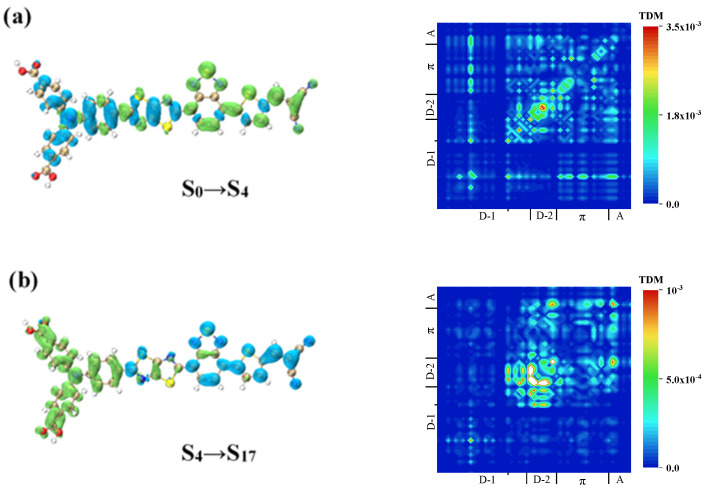

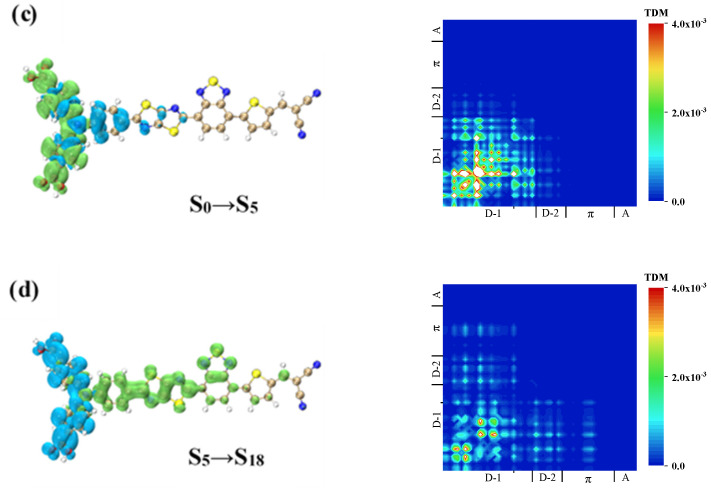
TDM and CDD of WS5 in TPA. TDM and CDD of S_0_→S_4_ (**a**) of WS5, TDM and CDD of S_4_→S_17_ (**b**) of WS5, TDM and CDD of S_0_→S_5_ (**c**) of WS5, TDM and CDD of S_5_→S_18_ (**d**) of WS5.

The absorption peak of WS5 was contributed to by S_17_ and S_18_. First, the excitation mode of S_0_→S_4_ of S_17_ was analyzed. It can be seen from the TDM in Figure 9a that triphenylamine underwent charge transfer with the π-linker and A. There was also charge transfer between the benzene ring on the inner side of triphenylamine and the inserted D (thiazolo[5,4-d]thiazole), and the two can be considered as a whole and also underwent charge transfer with the π-linker. The CDD shows the transfer of electrons from the D to the A, which was a charge transfer excitation on the whole.

As for the second step, S_4_→S_17_, it can be seen from Figure 9b that there was charge transfer between these four parts, among which the charge transfer between triphenylamine and the π-linker and A was relatively weak. The CDD showed a blue isosurface on the π- linker and A and a green isosurface on the triphenylamine, while the inserted D (thiazolo[5,4-d]thiazole) was blue-green, indicating that the electron transfer from the π-linker and A to thiazolo[5,4-d]thiazole and then to triphenylamine was a sequential charge transfer. The second process was the reverse transfer of electrons, which was opposite to the direction of charge transfer in the first process, indicating that the two-photon excited state of S_17_ was a locally excited state. The TDM and CDD of S_18_ are shown in Figure 9c and took S_5_ as the intermediate state. This was the same as the S_0_→S_1_→S_14_ excitation mode of W1 and was the local excitation enhanced charge transfer excitation.

**Figure 10:**
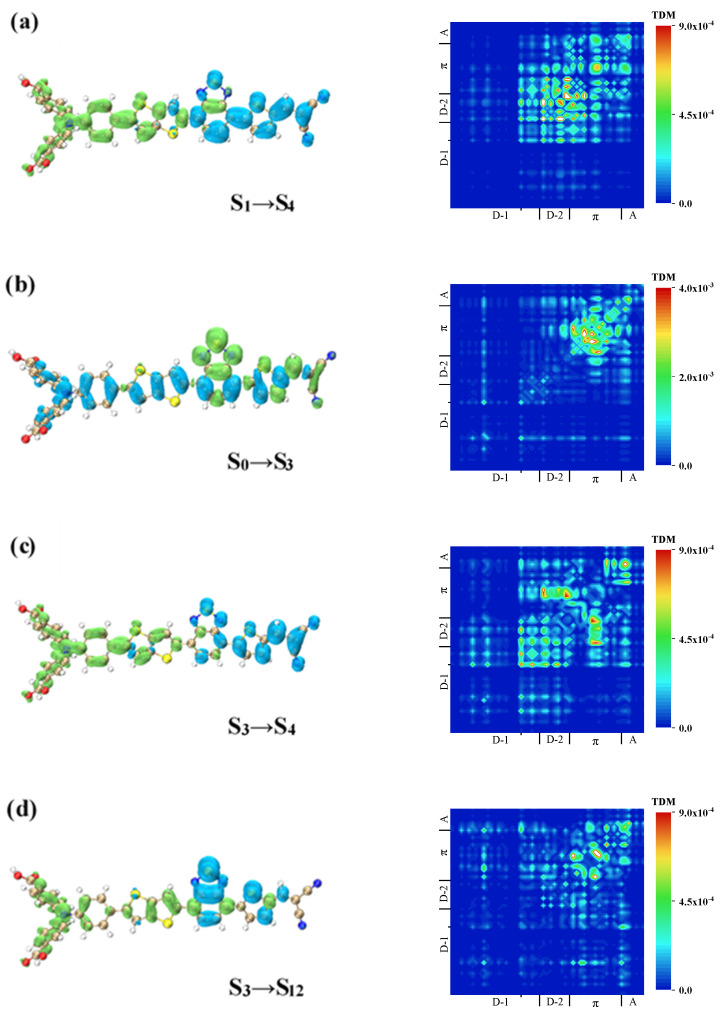

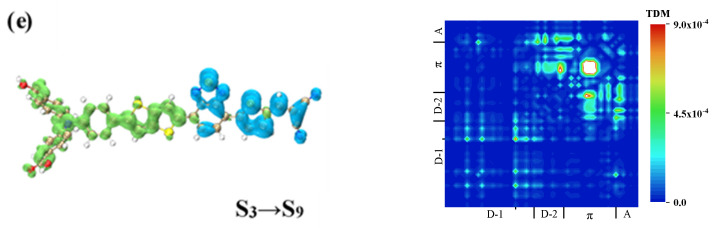
TDM and CDD of WS6 in TPA. TDM and CDD of S_1_→S_4_ (**a**) of WS6, TDM and CDD of S_0_→S_3_ (**b**) of WS6, TDM and CDD of S_3_→S_4_ (**c**) of WS6, TDM and CDD of S_3_→S_12_ (**d**) of WS6, TDM and CDD of S_3_→S_9_ (**e**) of WS6.

The TPA spectrum of WS6 had many absorption peaks, and its absorption range was relatively wide. S_12_ and S_9_, which constituted the strongest absorption peak, and S_4_, which had a larger absorption cross-section, were mainly analyzed. For the excited state of S_4_, it had S_1_ and S_3_ as intermediate states. According to the previous analysis, the first step of the first channel, S_0_→S_1_, was mainly the charge transfer in the A direction. The second process was the reverse transfer of electrons to triphenylamine, as shown in Figure 10a. The TDM indicated that the inserted D (bithiophene) not only had charge transfer with the π-linker, but also with the benzene ring on the inner side of triphenylamine. The CDD showed that the isosurface of the charge on the benzene ring on the inner side of triphenylamine was particularly large, and the isosurface of the inserted donor (bithiophene) was blue and green. Combined with the TDM, it was shown that electrons on the benzene ring were not only derived from the π-linker, but also from the bithiophene, and the electrons were transferred from the π-linker to the bithiophene and then to the benzene ring, the internal mechanism of which was the sequential charge transfer.

The first step of the other pathway was from S_0_ to S_3_. Both the π-linker and the A showed obvious local excitation and electron transfer excitation from the D to the A, as shown in Figure 10b. The second process of the second path was very similar to the first path in that electrons were transferred in reverse from the recipient to the donor, and the internal mechanism was sequential transfer, as shown in Figure 10c. By comparing the two paths, it was found that the excitation mode of the two paths was the same, and they were all local excitation on the whole, so the excited state was a local excitation.

It is noteworthy that the intermediate state of S_9_ and S_12_ was also S_3,_ and Table 4 shows their transition dipole moments. The transition dipole moment from S_3_ to S_12_ was relatively large, indicating that the transition probability of this process was relatively large. The CDD and TDM of the two processes of S_3_→S_12_ and S_3_→S_9_ are shown in Figure 10d,e. First, the S_3_→S_12_ process exhibited local excitation on the π-linker and A and generated charge transfer with the two donors, as shown in the TDM of Figure 10d. It can be demonstrated from the CDD that electrons were mainly transferred from the π-linker to the two donors, which was different from the sequential charge transfer of S_3_→S_4_. Another difference was that the hole isosurface on the A was relatively small, indicating a relatively weak degree of charge transfer.

For S_3_→S_9_, electrons were transferred from the π-linker and the A to the two D molecules, which was a complete charge transfer excitation, as shown in Figure 10e. For the three TPA excited states with S_3_ as the intermediate state, the charge transfer mode from S_3_ to each excited state was slightly different, but the charge transfer direction was the same. Because the second process of the three TPA excited states with S_3_ as the intermediate process was the reverse transfer of electrons, they were all charge local excitations. It can be said that the two-photon excited states with S_3_ as the intermediate process were all locally excited states.

**Figure 11:**
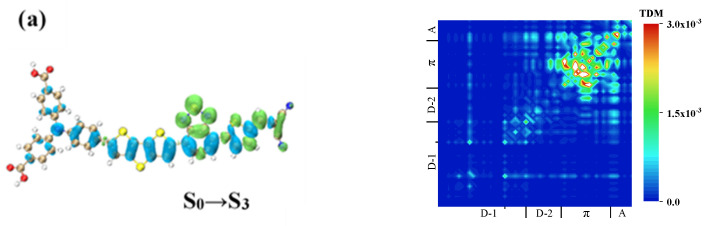

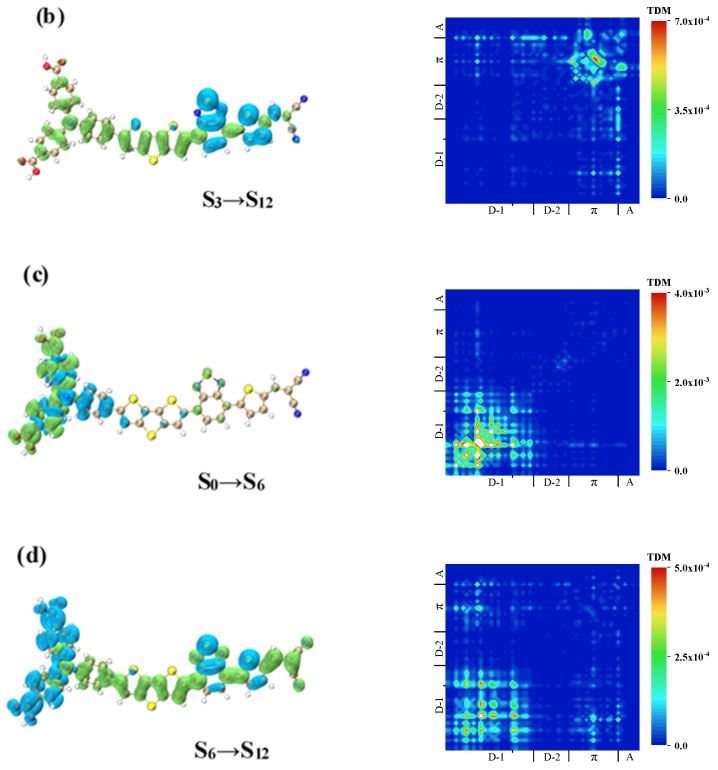
TDM and CDD of WS7 in TPA. TDM and CDD of S_0_→S_3_ (**a**) of WS7, TDM and CDD of S_3_→S_12_ (**b**) of WS7, TDM and CDD of S_0_→S_6_ (**c**) of WS7, TDM and CDD of S_6_→S_12_ (**d**) of WS7.

The strongest absorption peak of the WS7 molecule was contributed to by S_12,_ and the intermediate states with larger transition dipole moments were S_3_ and S_6_. First, we analyzed the first step, S_0_→ S_3,_ of the first path, where combining the TDM and CDD showed that the local excitation on the π-linker and acceptor was relatively strong, as confirmed in Figure 11a. The electrons on the π-linker and A came from two parts, one from the two benzene rings on the outer side of triphenylamine and the other from the benzene ring on the inner side of triphenylamine and the inserted D (s-DTT). The hole isosurface in this latter part was relatively large, indicating a strong degree of charge transfer. The TDM and CDD of the second step are shown in Figure 11b. As it can be seen, the electrons were mainly distributed on the two D molecules, and the holes were mainly distributed on the π-linker, indicating that the electrons on the two D molecules were transferred from the π-linker. The charge transfer directions of S_0_→ S_3_ and S_3_→ S_12_ were completely opposite, so the TPA excited state of S_12_ with the excited state of S_3_ as the intermediate state was a local excited state.

The first process of the other pathway, S_0_→S_7_, was the local excitation of the two benzene rings on the outer side of triphenylamine, where electrons were transferred from the benzene ring on the inner side of triphenylamine to the benzene ring on the outer side, as shown in Figure 11c. There were also electrons on the π-linker, proving from the TDM that electrons were transferred from triphenylamine. The TDM and CDD for this process are shown in Figure 11d. In the second process, electrons were transferred from the benzene ring on the outer side of triphenylamine to the benzene ring on the inner side, the π-linker, and the A. First, because S_0_→S_7_ was a local excitation, the whole TPA excited state was a local excitation enhanced charge transfer excitation. Second, there was also the charge transfer between the π-linker and S-DTT, which was opposite to the charge transfer direction on the π linker in the first step, but the degree of charge transfer was still relatively weak, so the excited state was dominated by local excitation enhanced charge transfer excitation. Comparing the charge transfer modes of these two paths is completely different, so the difference in the intermediate state may make the charge transfer mode of TPA completely different.

## 4. Conclusions

In this work, the OPA UV-vis spectra and TPA spectra of four dye molecules with triphenylamine as a donor were calculated and theoretically analyzed using density functional theory. First, the insertion of different donor groups increased the conjugated surface of the molecule and improved their light absorption intensity, resulting in a red shift in the UV-vis spectra. WS7 had the largest red shift and the largest light absorption range. Second, in the process of TPA, although the local excitation in the first process enhanced the charge transfer of W1, WS5, and WS7 in the second process, the internal transfer mechanism was also different. The insertion of the donor caused the D-D-π-A structure molecules to show the phenomenon of sequential charge transfer. Finally, the TPA cross-section of WS6 was relatively small compared to the other three, even smaller than that of the prototype W1, because its TPA process was mainly local charge excitation. Therefore, the insertion of different donors increased intramolecular charge transfer to different degrees and increased the TPA cross-section.

## Figures and Tables

**Table 1:** Excited state information for the first excited state of the four molecules.

Molecule	OPA States	*E*/ev	*λ*/nm	*f*
**W1**	S_1_	2.54660	486.86	1.59124
**WS5**	S_1_	2.39420	517.86	2.18762
**WS6**	S_1_	2.24780	551.58	1.98662
**WS7**	S_1_	2.17120	571.03	2.02813

**Table 2:** Transition dipole moments of the excited state S1 of W1, WS5, WS6, and WS7 (a.u.).

Molecule	x	y	z	Norm
**W1**	−5.047	−0.152	−0.006	5.050
**WS5**	6.105	0.135	−0.065	6.106
**WS6**	−6.003	0.130	−0.103	6.006
**WS7**	−6.129	−0.732	−0.157	6.174

**Table 3:** Contribution of fragment to the transition dipole moment of the excited state S_1_ (a.u.).

	**Part (D-1)**			**Part (D-2)**		
	**x**	**y**	**z**	**x**	**y**	**z**
**W1**	−0.728	−0.024	−0.009			
**WS5**	0.753	−0.023	−0.003	−0.028	−0.069	−0.058
**WS6**	−0.580	0.001	−0.014	0.065	0.022	−0.002
**WS7**	−0.580	0.026	−0.001	0.116	−0.419	−0.079
	**Part (** **π)**			**Part (A)**		
	**x**	**y**	**z**	**x**	**y**	**z**
**W1**	−0.277	−0.037	−0.000	−3.377	−0.228	−0.017
**WS5**	0.707	−0.000	−0.007	3.700	0.193	0.001
**WS6**	−1.010	0.021	0.042	−3.560	0.118	−0.130
**WS7**	−1.167	0.080	−0.024	−3.629	0.160	−0.033

**Table 4:** TPA element of the transition dipole moment matrix of four molecules.

Molecule	TPA States	Process	Integral Value
**W1**	S_14_	$<\varphi_{S_{0}}|\mu |\varphi_{S_{1}}>\to <\varphi_{S_{1}}|\mu |\varphi_{S_{14}}>$	25.50–1.91
		$<\varphi_{S_{0}}|\mu |\varphi_{S_{4}}>\to <\varphi_{S_{4}}|\mu |\varphi_{S_{14}}>$	6.41–2.48
	S_16_	$<\varphi_{S_{0}}|\mu |\varphi_{S_{9}}>\to <\varphi_{S_{9}}|\mu |\varphi_{S_{16}}>$	1.52–8.02
**WS5**	S_17_	$<\varphi_{S_{0}}|\mu |\varphi_{S_{4}}>\to <\varphi_{S_{4}}|\mu |\varphi_{S_{17}}>$	1.90–10.14
	S_18_	$<\varphi_{S_{0}}|\mu |\varphi_{S_{5}}>\to <\varphi_{S_{5}}|\mu |\varphi_{S_{18}}>$	6.29–5.47
**WS6**	S_4_	$<\varphi_{S_{0}}|\mu |\varphi_{S_{1}}>\to <\varphi_{S_{1}}|\mu |\varphi_{S_{4}}>$	36.07–33.15
		$<\varphi_{S_{0}}|\mu |\varphi_{S_{3}}>\to <\varphi_{S_{3}}|\mu |\varphi_{S_{4}}>$	5.94–8.79
	S_9_	$<\varphi_{S_{0}}|\mu |\varphi_{S_{3}}>\to <\varphi_{S_{3}}|\mu |\varphi_{S_{9}}>$	5.94–8.87
	S_12_	$<\varphi_{S_{0}}|\mu |\varphi_{S_{3}}>\to <\varphi_{S_{3}}|\mu |\varphi_{S_{12}}>$	5.94–21.04
**WS7**	S_12_	$<\varphi_{S_{0}}|\mu |\varphi_{S_{3}}>\to <\varphi_{S_{3}}|\mu |\varphi_{S_{12}}>$	6.69–2.23
		$<\varphi_{S_{0}}|\mu |\varphi_{S_{6}}>\to <\varphi_{S_{6}}|\mu |\varphi_{S_{12}}>$	5.23–1.61

## Data Availability

The data presented in this study are available on request from the corresponding author.
